# Recent advances and future perspectives of CAR-T cell therapy in head and neck cancer

**DOI:** 10.3389/fimmu.2023.1213716

**Published:** 2023-06-29

**Authors:** Chunmei Hu, Min Liu, Yutao Li, Yi Zhao, Amit Sharma, Haotian Liu, Ingo G. H. Schmidt-Wolf

**Affiliations:** ^1^ Department of Otolaryngology-Head and Neck Surgery, Sichuan Provincial People’s Hospital, University of Electronic Science and Technology of China, Chengdu, China; ^2^ Department of Otolaryngology-Head and Neck Surgery, West China Hospital of Sichuan University, Chengdu, China; ^3^ Department of Integrated Oncology, Center for Integrated Oncology (CIO), University Hospital Bonn, Bonn, Germany; ^4^ Department of Neurosurgery, University Hospital Bonn, Bonn, Germany

**Keywords:** CAR-T cell therapy, head and neck cancer, head and neck squamous cell carcinoma, immunotherapy, immune checkpoint inhibitors

## Abstract

Head and neck cancer (HNC) ranks as the sixth most prevalent type of cancer globally and accounts for about 4% of all types of cancer. Among all HNC, most are head and neck squamous cell carcinoma (HNSCC) with clinical therapies that include surgery, radiation therapy, chemotherapy, immunotherapy, targeted therapy, and multimodal treatments. In recent years, chimeric antigen receptor (CAR)-T cell immunotherapy has significantly transformed the therapeutic approaches for leukemia and lymphoma and has garnered increased attention as a potential treatment for a wide range of cancers. However, CAR-T immunotherapy in solid tumors, especially HNSCCs, lags significantly behind due to the paucity of tumor-specific antigens, high levels of tumor heterogeneity, immunosuppressive tumor microenvironment, the risk of treatment-related toxicities and off-target adverse events in HNSCCs. The objective of this review is to explore the advancement of CAR-T cell therapy in the treatment of HNSCCs. We aim to outline the targeted antigens in HNSCCs, highlight the challenges and potential solutions, and discuss the relevant combination therapies. Our review presents a comprehensive overview of the recent developments in CAR-T cell therapy for HNSCCs, and provides valuable insights into future research avenues.

## 1 Introduction

Head and neck cancer (HNC) is a cancer that develops in the facial, oral and neck regions, affecting aerodigestive tract, salivary glands and thyroid. HNC is the seventh most prevalent type of cancer globally (1) and accounts for about 4% of all types of cancer in the United States according to the cancer statistics published in 2021 (2). Tobacco and alcohol consumption are two of the most important risk factors that cause HNC. Human papillomavirus and Epstein-Barr virus infection are also risk factors linked to oropharyngeal cancer and nasopharyngeal cancer, respectively. Among all HNC, most are squamous cell carcinoma that derives from squamous cells of the mucosal epithelium in the facial, oral and neck regions, which are collectively known as HNSCCs. They exhibit immunosuppressive properties and are characterized by the presence of an inflammatory tumor microenvironment (3). Although clinical treatment for HNSCCs includes surgery, radiation therapy, chemotherapy, immunotherapy, targeted therapy, or multimodal therapy, the 5-year survival rate of patients with HNSCC has remained around 50% to 60% over the past few decades (4, 5).

Over the past few years, immunotherapy in cancer treatment has been steadily increasing, with numerous evidence supporting its valuable efficacy. For instance, Food and Drug Administration (FDA) granted approval for the use of Pembrolizumab, a humanized anti-programmed death receptor 1 (PD-1) antibody for the treatment of some patients with an advanced form of head and neck cancer in 2016 (6) whereas cetuximab, a monoclonal antibody that binds competitively to the EGFR, was approved by the FDA for late-stage (metastatic) and resistant cases of squamous cell carcinoma (SCC) of the head and neck in 2011 (7). Apart from PD-1, other immune checkpoints such as cytotoxic T-lymphocyte protein 4 (CTLA4), T-cell immunoreceptor with Ig and ITIM domains (TIGIT), and lymphocyte activation gene 3 protein (LAG3) are considered new rational therapeutic targets (8, 9). In addition, oncolytic viruses (OVs), antigenic vaccines, adoptive T cell transfer (ACT), costimulatory agonists therapy, are also under development (10). Although treatments for HNSCCs have improved consistently over the years, the prognosis of HNSCCs remains unsatisfactory, especially for recurrent and metastatic HNSCCs. Therefore, new treatments that can improve the chance of a complete cure and the prognosis of patients with HNSCCs are required.

**Figure 1:**
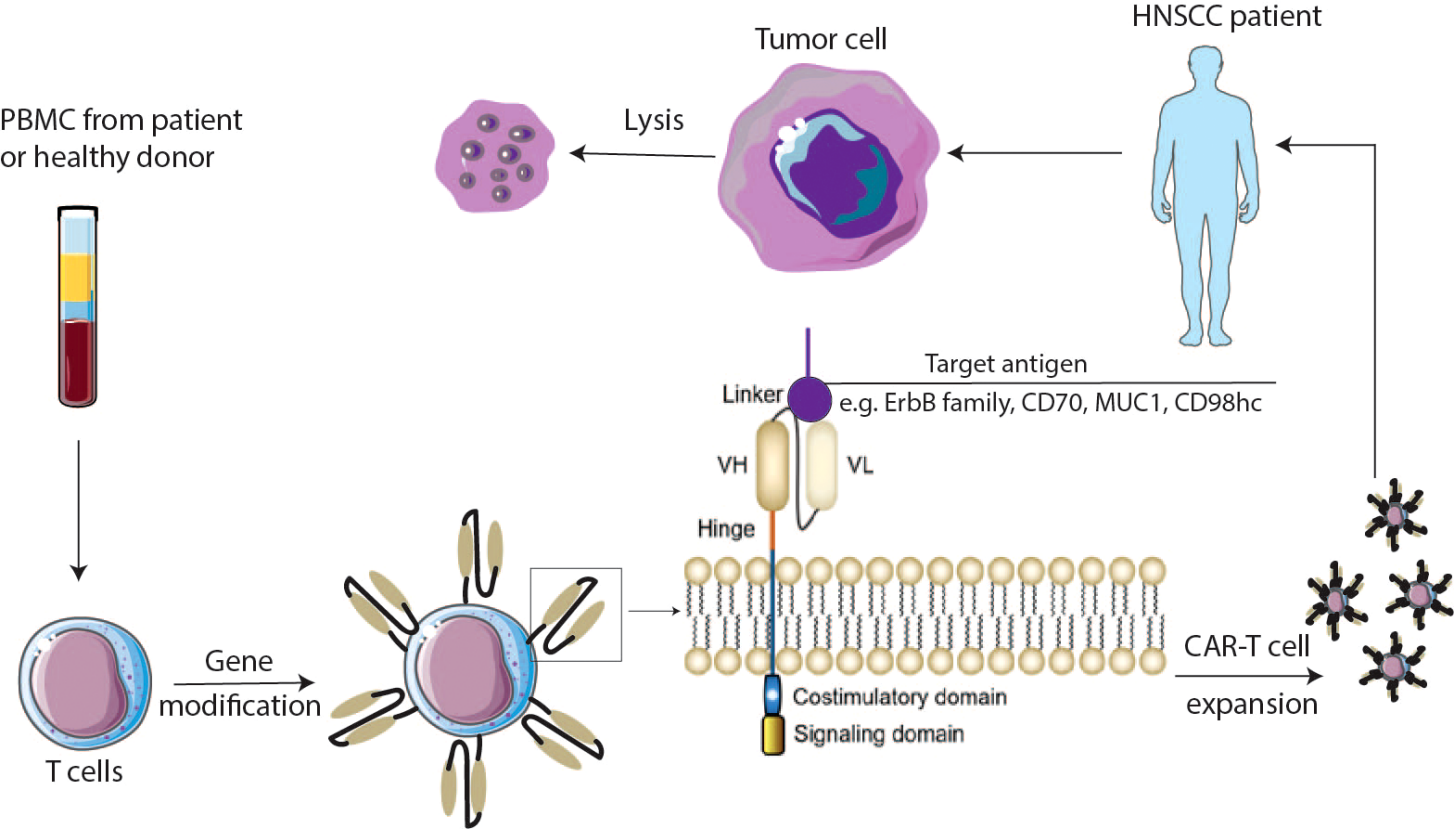
Schematic overview of CAR-T cells. PBMC cells are isolated from the patient’s or donor’s blood and expanded to T cells. Cells are genetically modified to express chimeric antigen receptors (CARs). CAR-T cells are expanded and injected into the HNSCCs patient’s body. CARs are composed of an antigen binding domain which is mainly derived from antibody, a hinge domain bridging between the antigen binding domain and the transmembrane domain, and the intracellular domains including costimulatory and signaling domains. VH—variable heavy chain; VL—variable light chain. CAR chimeric antigen receptor.

Chimeric antigen receptor T (CAR-T) cell therapy is a promising form of immunotherapy which has advanced to commercial approval for application in treating leukemia and lymphoma. To generate CAR-T cells, T cells obtained from either autologous or allogeneic T cells need to be modified to express a CAR (11, 12). With ongoing advancements in CAR-T structural design, CAR-T cells have progressed from their initial first generation to the current fifth generation (13). Typically, a CAR consists of mainly four components: (1) an antigen-binding domain, which is a single-chain variable antibody fragment that is in charge of recognizing tumor antigens (This is not the case for all CARs; for example, the CAR used in T4 immunotherapy does not have this domain); (2) a hinge domain, an extracellular structural segment that allows the extension of the antigen-binding domain from the transmembrane domain. Hinge domains help binding units gain access to target epitopes; (3) a transmembrane domain, which combines extracellular (antigen-binding domain and hinge domain) and intracellular structural domains; (4) an intracellular signaling domain, the part that has changed the most as CAR-T structural designing evolves. This domain encompasses both the signaling and co-stimulatory domain (the presence of the co-stimulatory domain is exclusive to the second and subsequent generations). Intracellular costimulatory molecular are often derived from receptors such as CD28, OX40, and CD137 (14, 15). CAR-T cells behave similarly to T cells, but CAR-T cells are capable of identifying and binding to specific targeted tumor associated antigens (TAAs) or tumor specific antigens (TSAs) on the surface of tumor cells through their specific antigen-binding domains, which initiates anti-tumor immune responses (**Figure 1**). Apart from this, CAR-T therapy offers a significant advantage over traditional immunotherapy due to its independence from MHC restriction, as immune evasion by cancer cells is mainly caused by a lack of MHC-associated antigen presentation (16). With the encouraging results of CAR-T therapy in treating hematological malignancies, more and more researchers are beginning to explore potential effects of this novel immunotherapy beyond solid tumors, including HNSCCs. Our work will review recent advancements of CAR-T therapy applied in HNSCCs, focusing on the targets of CAR-T cells, the challenges faced, and the corresponding engineering of CARs. Additionally, we will discuss the prospects for combination therapies to further enhance the efficacy of CAR-T therapy in the treatment of HNSCCs.

## 2 Target antigens of CAR-T cell therapy in HNSCCs

### 2.1 ErbB family

Among the potential targets for CAR-T therapy in HNSCCs, the ErbB family (also known as the epidermal growth factor receptor (EGFR) family), holds significant promise. This family is composed of EGFR, Human Epidermal Growth Factor Receptor 2 (HER2 or ErbB2), ErbB3 (HER3), and ErbB4 (HER4) (17, 18). All these receptors can be activated by binding to specific ligands. Upon this, the receptors form homo- or hetero-dimers, which activates their intrinsic tyrosine kinase activity and lead to multiple downstream signaling pathways that play a critical role in regulating cell growth, differentiation, survival, and migration.

The cytotoxic function of CAR-T cells targeted to EGFR against hypopharyngeal squamous cell carcinoma was verified as well in a preclinical study conducted by a team which had already successfully built and verified EGFR-CAR-T-cells (19). This research indicated a notable increase in cytokine secretion following the incubation of EGFR-CAR-T-cells with target tumor cells. This finding not only demonstrated the functional activity of these engineered T cells but also highlighted the enhanced cytotoxic effect specially against FaDu cells, a hypopharyngeal squamous cell carcinoma cell line. The EGFR-CAR-T cells exhibited a target cell lysis rate of 52.66%, further underscoring their potent therapeutic potential of treating HNSCCs.

In addition, T4 immunotherapy, a gene-modified immune cell treatment that specifically targets the ErbB homo- and heterodimers, has been suggested as a solution to the problems that CAR-T cell therapy encounters when treating solid tumors (20, 21). This immunotherapy is recommended because ErbB family members contributed significantly to the development of various types of cancer, including HNSCCs, and selectively targeting ErbB family members using monoclonal antibody therapy exhibits beneficial curative properties (22). It describes the engineering of patient T-cells by retroviral transduction to co-express a chimeric antigen receptor named T1E28z and a chimeric cytokine receptor named 4αβ (17). The T1E28z CAR has a promiscuous ligand of ErbB termed T1E that is connected to CD3ζ through a CD28 hinge/transmembrane and intracellular domain. Being positioned upstream of the CD28^+^ CD3ζ domain, T1E28z confers specificity on T cells against all EGFR homo- and heterodimers, the ErbB 2/3 heterodimer and all ErbB4 homo- and heterodimers. 4αβ chimeric cytokine receptor is created by fusing the ectodomain of IL-4 cytokine to the β chain used by IL-2 and IL-15. An ex vivo expansion system of CAR-T cells developed by Wilkie et al. suggested that a potent method for rapid proliferation of CAR-T cells is to cultivate them with IL-4 over a period of 7–10 days (23). One of the most challenging barriers of applying T4 immunotherapy is on-target off-tumor toxicity as ErbB family members are expressed in many healthy tissues. Pre-clinical evidence showed a possibility of reducing this toxicity by using the intra-tumoral route and a phase-I clinical study that recruited 13 patients who had been diagnosed with advanced HNSCCs has been developed and demonstrated the safety of intra-tumoral T4 (24). The whole trial is still under recruitment and expected to be completed by 2023 (NCT01818323) (25).

### 2.2 CD70

Although CD70 expression was high in 19% of HNSCC tumor biopsies, Park et al. utilized a retroviral human CD70 CAR construct to generate CD70 CAR-T and found that it presented the capacity of efficiently eliminating CD70-positive HNSCC cells, which might be related to the transduction efficiency, the CD4^+^/CD8^+^ T cell ratio, and a different response to IL-2 stimulation. Consistently, the infusion of CD70-targeted CAR-T cells into patients with clear-cell renal carcinoma showed an outcome of a disease control rate of 76.9%. Among the 14 patients enrolled, one patient had a long-lasting full remission that is still going on as of their 2-year follow-up (26). This outcome further encourages the feasibility of CD70-targeted CAR-T therapy for CD70-positive HNSCC patients.

### 2.2 MUC1

The MUC1 gene was discovered in human breast cancer with high expression. It is expressed in barrier epithelia lining (27) and skin, immune cells and reproductive organs (28). Therefore, it plays multiple roles in protecting barrier epithelia as well as species propagation in mammals (27). It has been reported that CAR-T cells targeting the tumor MUC1 inhibit triple-negative breast cancer growth in murine models whereas there is minimal damage to normal breast epithelial cells (29). Furthermore, it has been demonstrated that MUC1 has a higher expression in HNSCCs than in adjacent normal tissues in a multivariate analysis (30). Since there was evidence that IL-22 contributed to inducing an elevation of MUC1 expression in colon cancer patients (31), Mei et al. constructed MUC1‐IL22 CAR-T cells and found they exerted more effective cytotoxic function than MUC1 CAR-T cells *in vitro* and *in vivo* (3).

### 2.3 CD98hc

CD98hc, which is a strong related to T cell activation, has been proved to be highly expressed on the surface of radioresistant HNSCC cells (32). CD98hc-targeted UniCAR-T cell was developed and showed ideal ability of tumor cell elimination in a 3D setting of HNSCCs tumor spheroid (33). In addition, the infiltration capability of the UniCAR T cells was improved in the presence of the CD98hc targeted system and a synergistic effect was observed after treatment with fractionated irradiation followed by CD98hc-redirected UniCAR-T delivery.

Apart from these published results, several clinical trials of CAR-T therapy for the treatment of patients with head and neck cancer are underway, targeting novel antigens such as EpCAM, LAMP1, PSMA. We summarized in **Table 1**.

**Table 1:** Summary of ongoing CAR-T therapy with different target antigens for head and neck cancer on US Clinical Trials Register (clinicaltrials.gov) or EU (clinicaltrialsregister.eu).

NCT	Study Title												
	Origin	Antigen		Cancer		Phage		Start		No. patients		Vector	Sponsors
01818323	Phase I Trial: T4 Immunotherapy of Head and Neck Cancer.												
(24, 25)	Autologous	ErbB		HNSCC		I		2015		30		RV, IT	King’s College London, U.K.
02915445	T Cells Armed With Chimeric Antigen Receptor Recognizing EpCAM for Patients With Advanced Solid Tumors.												
	Autologous	EpCAM		nasopharyngeal carcinoma, breast cancer		I		2016		30		LV, IV	Sichuan University, China
02980315	A New Efficient EBV AssociatedTechnologies of T Cells in Treating Malignant Tumors and Clinical Application.												
	Autologous	LMP1		EBV^+^ solid tumors		I/II		2016		20		LV, IV	Nanjing Medical University, China
03013712	A Clinical Research of CAR T Cells Targeting EpCAM Positive Cancer.												
	Autologous	EpCAM		EpCAM^+^ solid cancers		I/II		2017		60		LV, IV	Chengdu Medical College, China
04107142	A Phase I Dose-escalation Trial to Evaluate Haploidentical/Allogeneic Natural Killer Group 2D Ligand (NKG2DL)-Targeting Chimeric Antigen Receptor-grafted Gamma Delta (γδ) T Cells (CTM-N2D) in Subjects With Relapsed or Refractory Solid Tumor.												
	Allogeneic	NKG2DL		relapsed or refractory solid tumor		I		2019		10		LV, IV	CytoMed Therapeutics Pte Ltd., Malaysia
04249947	A Phase 1 Dose Escalation and Expanded Cohort Study of P-PSMA-101 in Subjects With Metastatic Castration-Resistant Prostate Cancer (mCRPC) and Advanced Salivary Gland Cancers (SGC).												
	Autologous	PSMA		mCRPC, SGC		I		2020		60		LV, IV	Poseida Therapeutics, Inc., U.S.A.
04420754	A Multicenter Phase I Study of AIC100 CAR T Cells in Relapsed and/or Refractory Advanced Thyroid Cancer or Anaplastic Thyroid Cancer.												
	Autologous	ICAM-1		relapsed/refractory thyroid cancer		I		2020		24		LV, IV	AffyImmune Therapeutics, Inc., U.S.A.
03740256	A First in Human Phase I Trial of Binary Oncolytic Adenovirus in Combination With HER2-Specific Autologous CAR T Cells in Patients With Advanced HER2 Positive Solid Tumors.												
	Autologous	HER2		HER2^+^HNSCC or solid tumors		I		2020		45		CAdVEC, IT	Baylor College of Medicine, U.S.A.
04877613	Phase I Trial of GFRα4 CAR T Cells in Adult Patients With Recurrent or Metastatic Medullary Thyroid Cancer.												
	Autologous	GFRα4		recurrent or metastatic medullary thyroid cancer		I		2021		18		LV, IV	University of Pennsylvania
05239143	A Phase 1 Dose Escalation and Expanded Cohort Study of P-MUC1C-ALLO1 in Adult Subjects With Advanced or Metastatic Solid Tumors.												
	Allogeneic	MUC1-C		advanced or metastatic solid tumors		I		2022		100		SB, IV	Poseida Therapeutics, Inc., U.S.A.
04847466	A Phase II Study of Immunotherapy Combination: Irradiated PD-L1 CAR-NK Cells Plus Pembrolizumab Plus N-803 for Subjects With Recurrent/Metastatic Gastric or Head and Neck Cancer.												
	Autologous	PD-L1		recurrent/metastatic HNC		II		2021		55		LV, IV	National Cancer Institute (NCI), U.S.A.
EudraCT 2019-004323-20	Phase 1/2a, first-in-human, open-label, dose escalation trial with expansion cohorts to evaluate safety and preliminary efficacy of CLDN6 CAR-T with or without CLDN6 RNA-LPX in patients with CLDN6-positive relapsed or refractory advanced solid tumors.												
	Autologous	CLDN6		CLDN6^+^ relapsed advanced solid tumors		I/IIa		2020		18		RV, IV	BioNTech Cell & Gene Therapies GmbH, Germany

Antigen: EpCAM, epithelial cell adhesion molecule; LMP1, latent membrane protein 1; NKG2DL, natural killer group 2; PSMA, prostate-specific membrane antigen; HER2, human epidermal growth factor receptor 2; MUC1-C, mucin 1-C; ErbB, erythroblastic oncogene B; ICAM-1, intracellular adhesion molecular-1; HER2, human epidermal growth factor receptor 2; GFRα4, glial cell line-derived neurotrophic factor receptor alpha; MUC1-C, mucin 1-C. Cancer: HNSCC, Head and Neck Squamous Cell Carcinoma; mCRPC, metastatic Castration-Resistant Prostate Cancer (mCRPC); SGC, Advanced Salivary Gland Cancers; Head and neck cancer, HNC. Vector: LV, lentiviral vector; RV, retroviral vector; SB, sleeping beauty; IV, Intravenous infusion; IT, Intratumoral injection.

## 3 Overview of challenges of CAR-T cell immunotherapy in the treatment of HNSCCs

### 3.1 Paucity of tumor specific antigens and high levels of tumour heterogeneity in HNSCCs

Human tumor antigens can be classified into viral antigens, neoantigens that are derived from new, mutated genomic sequences, tumor-specific antigens (TSAs) that derive from unmutated proteins and that are either unique or specific for tumors, and tumor-associated antigens (TAAs) that are differentially expressed in tumor cells and less so in normal cells (34). The optimal target antigens for CAR-T therapy are those tumor-specific antigens (TSAs) exclusively expressed on malignant cells. Specific tumor-specific antigens (TSAs) are neoantigens presented on major histocompatibility complex (MHC) molecules that are suitable for personalized cancer vaccines instead of general CAR-T cell therapy (35). However, these antigens are highly heterogeneous among patients suffering from the same type of tumors, which causes challenges for CAR-T cell therapy. Schumacher, et al. suggest cancer exome-based identification of neoantigens for each patient and assess T cell recognition before generating CAR-T cells (36). Compared to hematological malignancies, targeting TAAs in solid tumors elicits stronger on-target off-tumor effects (37, 38).

When these two antigens can`t achieve satisfactory outcomes *in vitro*, other options for choosing target antigens for CAR-T might be considered. One alternative antigen is fibroblast activation protein (FAP) in HNSCCs. A novel strategy of CD40 agonist targeted to FAPα combined with radiotherapy has been proven to remodel the TME, activate the CD8^+^ T cells without the systemic toxicity in murine HPV-positive head and neck tumors. This model increased the overall survival and induced long-term antitumor immunity (39). A previous phase I clinical trial of malignant pleural mesothelioma locally treated with autologous anti-FAP-targeted CAR T-cells (NCT01722149) has been reported that no significant toxicity was observed and the activity of the patient’s redirected T cells was confirmed *in vitro* (40). Therefore, FAP-targeted CAR-T cells combined with other immune therapies could be a potential strategy for HNSCC patients.

### 3.2 Inefficient CAR-T cell trafficking in the treatment of HNSCCs

The access of CARs to tumors and their infiltration into the tumor microenvironment (TME) are prerequisites for successful therapies. As with other solid tumors, treatment of HNSCCs must overcome two main barriers: the vascular and stromal barriers. Remodeling of the vascular system hinders immune cells’ ability to penetrate blood vessels to reach the tumor site (41). In the meantime, the stromal barrier of solid tumors consisting of abnormal extracellular matrix (ECM) or mismatched chemokine receptors of immune cells and tumor-derived chemokines also blocks T cell migration (35). Cancer-associated fibroblasts (CAF) within the TME facilitate the accumulation of abnormal ECM around the tumor, eventually creating a compact fibrotic environment. Activating TGF-β can trigger CAF to release extra ECM proteins, limiting T cell’s mobility. Therefore, researchers suppress TGF-β signaling and CAF differentiation, reducing EMC protein secretion and eventually encouraging immune cell infiltration into tumor tissues by inhibiting NOX4, a downstream molecule of TGF-β signaling (42). In the study of Trastuzumab-derived HER2-specific CARs for treating trastuzumab-resistant breast cancer, CAR-T cells can infiltrate the tumor matrix and eliminate tumors that are not accessible to antibodies (43).

In addition, Heparanase (HPSE) can degrade heparin sulfate proteoglycan in the ECM. CAR-T cells expressing HPSE were therefore designed to decompose the ECM and improve T cell infiltration and antitumor efficacy (44). In order to enhance CAR-T cell trafficking, direct injection of CAR-T cells into the tumor site could prevent cells’ circulation through the vascular transport system. For instance, in a study of IL13Rα2-targeted CAR-T cells against glioblastoma, local intracranial delivery of CAR T cells displayed superior anti-tumor efficacy as compared to intravenous administration (45). A similar scenario was reported in murine models with atypical teratoid/rhabdoid tumors (ATRTs) in the treatment with B7-H3-targeted CAR-T cells either intracerebroventricularly (ICV) or intratumorally (IT) compared to intravenously (IV) (46). Even though the lack of clinical lymphodepletion in murine models and the functional distinction between murine and human B7-H3, evidence of intratumaral delivery of CAR-T from animal experiments might provide fundamental data to support clinical trials.

A novel approach for regional delivery of CAR-T cells to solid tumors has also been reported when using implantable biopolymer scaffolds to eliminate heterogeneous tumors. The concurrent transfer of CAR-T cells and the stimulator of INF genes (STING) agonist has the potential to enhance the immune response to tumor cells that do not express the CAR-specific target molecule while locally providing growth factors to CAR-T cells can protect them from the hostile TME during the initial phase of tumor priming (47).

### 3.3 Immunosuppressive tumor microenvironment in the treatment of HNSCCs

Various immunosuppressive substances and cells that decrease the elicited antitumor immunity are produced and recruited in TME by cancer cells, cancer-associated fibroblasts, and stromal cells (48). In a previous study, the locomotion of CAR-T cells in the solid tumor microenvironment was carefully addressed (49).

Local delivery of cytokines and chemokines by CAR-T cells within the TME, such as IL-22, IL-18, IL-12, and the combination of CCL19 and IL-7, or checkpoint blocking agents, can help in overcoming barriers to T cell infiltration and functioning (50). Besides the exogenous addition of IL-22, CAR-MUC1-IL22 T cells, the fourth-generation CAR-T cells that could release the IL-22 cytokine, may also boost recognition and cytotoxic potential against HNSCC (3).

T-cell exhaustion is significantly influenced by the activation of the transcription factor family NR4A, which is connected to the production of immunosuppressive molecules in solid tumors such as PD-1 and TIM3. CAR-T cells can exhibit better tumor-killing activity and enhanced persistence with the knockout of NR4A (51).

### 3.4 Treatment-related toxicities in the treatment of HNSCCs

Toxicities associated with CAR-T cell therapy can be classified into two major categories. (1) “On-target off-tumor” toxicity results from specific interactions between the CAR and its target antigen expressed by non-malignant cells. (2) general toxicities, which are related to T cell activation and subsequent systemic release of high levels of cytokines, including cytokine release syndrome (CRS), immune effector cell-associated neurotoxicity syndrome (ICANS), hemophagocytic lymph histiocytosis (HLH), and/or macrophage activation syndrome (MAS). ICANS and CRS are the two most severe and unanticipated reactions to CAR-T cell therapy within the category of general toxicities, as reported in the literature (52). CRS, a systemic inflammatory response, is the most common adverse effect caused by CAR-T cell therapy. The most prevalent symptom of CRS following CAR-T cell infusion is fever, which can be accompanied by nausea, fatigue, hypotension, and cardiac dysfunction (53). ICANS is linked with the disturbance of the blood-brain barrier and the rise of cytokine levels in the cerebrospinal fluid. Usually, ICANS arise alongside or after CRS (54). The clinical signs of ICANS linked to CAR-T cell therapy cover encephalopathy, memory impairment, seizures, speech impairment, tremors, headaches, language disturbance, and motor weakness (55, 56). In contrast to CRS, the exact underlying pathophysiology of ICANS is currently vaguely comprehended (57).

Several strategies have been developed to address the challenge of toxicity, such as multitarget CARs, affinity-optimized CARs, inhibitory CARs (iCARs), incorporation of suicide genes into CAR-T cells, and utilization of transient RNA-expressing CARs (58). Kosti et al. developed a CAR-T cell capable of detecting hypoxia. This approach relies on the expression of pan-ErbB-targeted CARs in the hypoxic site. The results demonstrate that CAR molecule expression can only be detected in the hypoxic tumor site of HNSCC, while it is undetectable in normal tissues (59).

## 4 Therapeutic strategies to enhance CAR-T cells

### 4.1 Combinatorial antigen targeting

#### 4.1.1 Multiple CARs with “OR” strategy

“OR” strategy refers to immunotherapies with multiple CARs that could be (1) Combined CAR-T cell immunotherapy, which refers to the simultaneous or sequential use of two or more CAR-T cells to treat a similar malignant tumor. This pooled mixture of two populations of CAR-T cells is also referred to as CARpool, (2) co-expression of two to three distinct CARs in individual T cells (dual CAR, triple CAR), or (3) tandem CARs (TanCAR) which are formed by two different scFvs linked together. The “OR” strategy boosts the density of the targetable molecules on the tumor surface, thereby augmenting CAR-T cell potency and antitumor efficacy. For instance, the effective combinatorial application of CAR-CD19-CD28-T cells and CAR-CD19-4-1BB-T cells demonstrates how the Trogocytosis of CAR-T cells may be counteracted with the “OR” Strategy through the CARpool method (60). Nanobodies, heavy chain antibodies found naturally in sharks and camelids, have recently been suggested as scFv substitutes and may enable the production of compact CARs that possess dual specificity and predetermined affinity (61).

#### 4.1.2 CAR-T cells secreting bispecific t-cell engagers (BiTEs)

CAR-T cells can be designed by endogenous, non-engineered T cells through the secretion of BiTEs, which consist of two scFvs, one specific to CD3 and the other to a TAA, connected by a flexible linker. Studies suggest that BiTE-secreting CAR T cells can target multiple antigens, possess better antitumor activity than single-target CAR-T cells, and can prevent tumor escape resulting from antigen heterogeneity while activating and recruiting bystander T cells (42, 62–65). Hence, BiTE-secreting CAR T cells could be a promising therapeutic strategy for HNSCCs.

#### 4.1.3 Universal CARs

The fifth-generation CARs also called universal CARs, employ adapter elements as ligands that enable the targeting of multiple antigens with a distinct CAR-T cell population. Besides, the SUPRA (split, universal, and programmable) CAR system, which consists of a leucine zipper CAR (zipCAR) and an scFv binding to the leucine zipper (zipFv), is only active when zipFv are present, enabling targeting of multiple antigens as well as reducing potential immunogenicity (66).

#### 4.1.4 Multiple CARs with “AND” strategy

The “AND” strategy enhances the safety of CAR-T cells by reprogramming them to activate only in response to target cells expressing two antigens concurrently, resulting in an increased specificity.

Wilkie et al. first hypothesized that CAR-mediated CD3ζ signaling 1 is responsible for the cytotoxicity and IFN-γ production, while the additional CD28-mediated signal 2 promotes T-cell proliferation and IL-2 production. To test it, dual T cells were engineered to co-express ErbB2 and MUC1 with the signaling domains of CD3ζ and CD28 respectively. To test it, dual T cells were engineered to co-express ErbB2 and MUC1 with the signaling domains of CD3ζ and CD28 respectively. This dual CAR-T displayed ErbB2-dependent cytotoxicity and MUC1 and ErbB2-codependent proliferation. However, production of IL-2 was modest compared to control CAR-T containing CD3ζ and CD28 fused domains (67). These findings demonstrate that dual-target CAR-T with cytotoxic and proliferative functions is achievable *in vitro*.

In addition, Wittsten et al. and Choe et al. developed the synthetic notch (synNotch) “AND” Gate T Cells, capable of inducing target-gene expression upon detecting a cell surface-bound ligand. SynNotch can identify a TAA and stimulate the production of a CAR, which can then activate T-cells upon identification of a second TAA (68). According to research, using synNotch-CAR T cells could potentially serve as a viable approach for solid tumors, decreasing systemic toxicity compared to constitutive CAR expression, as long as the off-tumor target is not near the tumor cells (69, 70).

**Figure 2:**
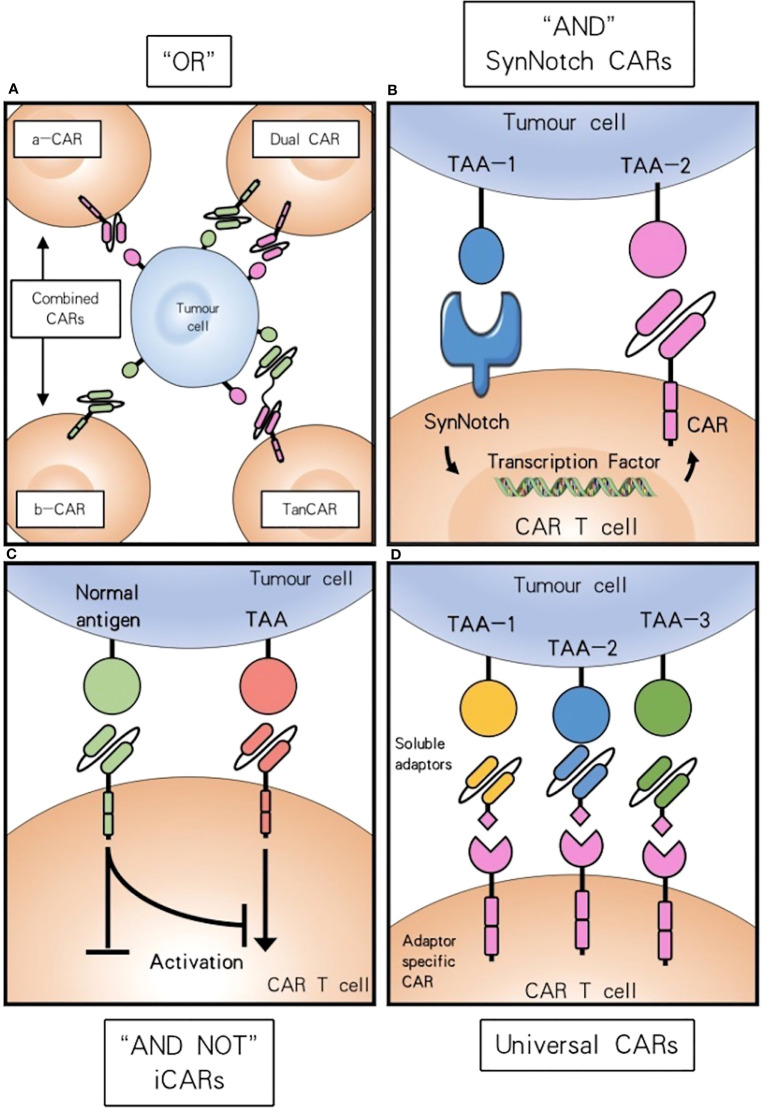
Combinatorial antigen targeting for HNSCCs. **(A)** For “OR” gate the presence of one antigen is sufficient to trigger effector function, while concurrent expression of both antigens leads to synergistical improvement of activation. **(B)** “AND” gate SynNotch CAR T cells require the presence of either target antigens to efficiently activate. **(C)** Combinatorial antigen recognition by AND-NOT logic using an iCAR can increase antigen specificity and safety. **(D)** Universal CAR T cells can target a variety of different antigens since their antigen specificity comes from the administration of soluble adaptors.

CAR-T cells performing AND-NOT logic can mitigate adverse effects on benign cells. The approach employs an inhibitory chimeric antigen receptor (iCAR) that selectively recognizes an antigen presented in healthy tissue, combined with an activating CAR that targets a TAA (71). (**Figure 2**).

### 4.2 Increase Success of CAR-T by Chemotherapy and Radiotherapy

The manufacturing process for CAR-T takes about three to four weeks, which can be a fatal waiting time for patients whose disease might continue to be aggressive. Adding bridging chemotherapy prior to CAR-T is currently regarded to be an essential to eradicate tumor cells and boost the efficacy of CAR-T cell therapy in hematologic malignancies (35, 72) Furthermore, the combination of CAR-T therapy with chemotherapy reduces immune suppressor cells in the TME and enhances T cell trafficking to tumors with upregulation of chemokines including CCL5, CXCL9 and CXCL10 (73). However, the combination of chemotherapy with CAR-T cell therapy poses challenges. For instance, the susceptibility of naïve T-cells towards chemotherapy may decrease CAR-T cell efficacy. Besides, chemo-agent cisplatin *via* HSF1-HSP90 axis enhances the expression of functional PD-L1 in oral squamous cell carcinoma (74). Therefore, the combination effects may be dependent on the dose and the treatment schedule (73).

Radiation therapy (RT) also plays a vital role in the management of HNSCCs and is the primary treatment for nasopharyngeal carcinoma. Radiation modulates tumor immune microenvironments *via* the release of inflammatory mediators to attract and mediate immune cells. On the other hand, TAAs produced by irradiated tumor cells can be captured by antigen-presenting cells (APCs) (75). Furthermore, activated T cells circle and sequentially have an effect on unirradiated metastases, which is called the abscopal effect (76). With the increasing use of CAR-T, synergistic effects highlight a potential strategy. Further preclinical evidence has been described that NKG2D-based CAR-T cells in mouse glioma models demonstrated efficacy, long-term persistence, and synergistic activity in combination with radiotherapy (77).

Although curative radiotherapy is challenging as a subset of patients are radioresistant (78), combination of conventional radiotherapy with CAR-T therapy turns out encouraging outcomes. Mechanically, RT delivered after CAR T cells resulted in expansion of TCR repertoires, which is an abscopal-like response in a case of a patient with multiply relapsed, refractory myeloma (79). Low-dose RT delivered before CAR T cells supports CD19-targeting CAR T cells in a murine leukemia model (80). In particular, Köseer et al. constructed a 3D culture system of HNSCCs tumor spheroid and demonstrated that the antitumor effect of a CD98hc-retargeted UniCAR-T system was not impaired by irradiation (33). From fractionated irradiation followed by UniCAR-based immunotherapy in Cal33 radioresistant spheroids, they observed that increased UniCAR T expansion with increased EGFP signal and enhanced UniCAR T function with increasing granzyme B and IFN-γ after this combination therapy, indicating an intriguing strategy for the therapy of patients with high-risk HNSCCs (32).

### 4.3 Improving the efficacy of CAR-T by mRNA vaccine

The feasibility of therapeutic mRNA vaccination against cancer was first demonstrated in 1995 (81). Since then, plenty of studies on mRNA-pulsed DC vaccines have emerged. There are two main mRNA-based immunotherapy approaches. The first strategy is to directly deliver mRNA, involving naked mRNA or mRNA complexed with lipid nanoparticle (LNP), by different administration routes (local injection, such as intramuscular, subcutaneous, and intradermal injection, are major routes for mRNA vaccines in clinical studies). The latter strategy is based on the administration of mRNA-modified DCs or chimeric antigen receptor (CAR) T cells. mRNA vaccines have been utilized in the treatment of aggressive and less accessible solid tumors, however, DC mRNA vaccines are determined by multiple factors. mRNA-encoded proteins are processed to antigenic peptides that associate with MHC class I molecules targeting CD8^+^ T cells only, not CD4^+^ T helper cells, to boost antitumor responses. Additionally, posttranscriptional modifications of peptide epitopes (82, 83) and the duration of the antigen peptide-loaded DC presented, DC-cell density, injection site might be considered.

A recent study documented that Reinhard et al. introduced the developmentally regulated tight junction protein claudin 6 (CLDN6) as a CAR target in solid tumors and demonstrated that CAR-T cell-amplifying RNA vaccine was a suitable approach in treating with CLDN6^+^ lung tumors, without cytokine release syndrome (CRS) (84). Furthermore, this RNA vaccine is efficient in *in vivo* proliferation and in improving CAR-T cell persistence. Based on data from the Cancer Genome Atlas (TCGA), CLDN6 expression was significantly upregulated in head and neck squamous cell carcinoma (HNSC) (85). Recently, the ongoing clinical trial (EudraCT No. 2019-004323-20) holds a promising future in using CAR-T cell immunotherapy to treat HNSC patients although barriers including high tumor heterogeneity and the maintenance of T cell immune responses in the TME have yet to be addressed.

### 4.4 Boosting CAR-T by immune checkpoint inhibitors or co-stimulatory molecules

It is well known that immune checkpoint molecules interactions with their ligands prevent recognition between T cells and antigen-presenting cells (86–88). PD-1 and CTLA-4 display distinct functions since they utilize different structural motifs to bind and recruit phosphatases for signal blockade. Blocking of PD-1-PD-L1 but not CTLA-4-B7 interactions enhances tolerized T cell interactions with antigen-bearing DCs and facilitates the phosphorylation of TCR signaling molecules (89). In an orthotopic mouse model of pleural mesothelioma, high doses of both CD28- and 4-1BB-based second-generation CAR T cells eradicated tumors effectively, whereas PD-1 upregulation in the tumor microenvironment inhibited T cell function. Using PD-1 antibody, PD-1 shRNA blockade, or a PD-1 dominant negative receptor restored the function of CD28 CAR-T cells (90). Clinically, a recent phase I clinical study conducted by Adusumilli et al. provided satisfactory median overall survival results for using pembrolizumab and CAR-T together when treating patients with malignant pleural disease (91). An alternative method of targeted delivery of a PD-1-blocking scFv by CAR-T cells not only enhanced the anti-tumor efficacy of CAR-T but also improved bystander tumor-specific T cells in xenogeneic mouse models of PD-L1^+^ hematologic and solid tumors (92). Clinically, a recruiting phase I trial of T4 immunotherapy of head and neck cancer (NCT01818323) is designed to use panErbB-specific CAR T cells intratumorally and involves the administration of nivolumab. This clinical trial might provide fundamental evidence for the toxicity and persistence of CAR-T combined PD-1 inhibitor. Interestingly, Lee et al. evaluated CD19-targeting CAR-T cells in the context of four different checkpoint combinations-PD-1/TIM-3, PD-1/LAG-3, PD-1/CTLA-4, and PD-1/TIGIT-and found that CAR T cells with PD-1/TIGIT downregulation exerted synergistic antitumor effects while dual downregulation of PD-1/LAG-3 severely damaged antitumor activity. Mechanically, downregulation of PD-1 enhances short-term effector function, whereas downregulation of TIGIT is primarily responsible for remaining a less exhausted state. The efficacy and safety of PD-1/TIGIT-downregulated CD19-targeting CAR-T cells are being assessed in patients with large B cell lymphoma (NCT04836507) (93). The utilization of dual immune checkpoint blockades might provide novel perspectives for the treatment of patients with solid tumors in CAR-T therapy. However, it is vital to carefully monitor the potential risk such as CRS and ICANS.

Conversely, CD80/CD86-targeted CAR-T (CTLA4-CAR), which comprises the extracellular and transmembrane portions of human CTLA4, the cytoplasmic region of human CD28, and the intracellular domains of human CD3z, was designed to destroy CD80/CD86-associated tumor cells *in vitro* and vivo. CTLA4-CAR T cells promoted IL-2 and IFN-γ secretion and suppressed tumor growth in xenograft models of malignant B cells. Furthermore, CTLA4-CAR T cells were found to infiltrate in residual tumors and are cytotoxic to myeloid-derived suppressor cells (MDSCs) without signs of severe CRS in murine models (94). Similarly, CTLA-4 tail fusion has superior anti-tumor efficacy in a relapsed leukemia model (95).

Apart from immune checkpoints, co-stimulatory members of the TNFRSF molecule (4-1BB, OX40, CD27, CD40, HVEM, and GITR) display unique functional roles in CAR-T therapy for solid tumors. CD40 ligand-modified CAR T cells displayed superior antitumor efficacy, promoted upregulation of co-stimulatory markers CD80 and CD86 on CD40^+^ lymphoma cells, educated antigen-presenting cells, enhanced recruitment of immune effectors, and mobilized specific tumor-recognizing T cells without outward signs of toxicity (96). Mata et al. introduced an inducible costimulatory (iCO) molecule consisting of a chemical inducer of dimerization (CID)-binding domain and the MyD88 and CD40 signaling domains into CAR-T and found that the CAR-T cells improve the efficacy of CAR T-cell therapy for solid tumors (97). Golubovskaya et al. designed CAR with a glucocorticoid-induced TNFR-related protein (GITR) co-stimulatory domain and recorded that EGFR-GITR-CD3 CAR-T cells were cytotoxic against EGFR^+^ pancreatic and ovarian cancer cells with higher levels of IFN-γ (98). These results suggest co-stimulatory molecules could be a potential option to enhance the activity of CAR-T in HNSCCs.

### 4.5 Oncolytic adenoviruses

**Figure 3:**
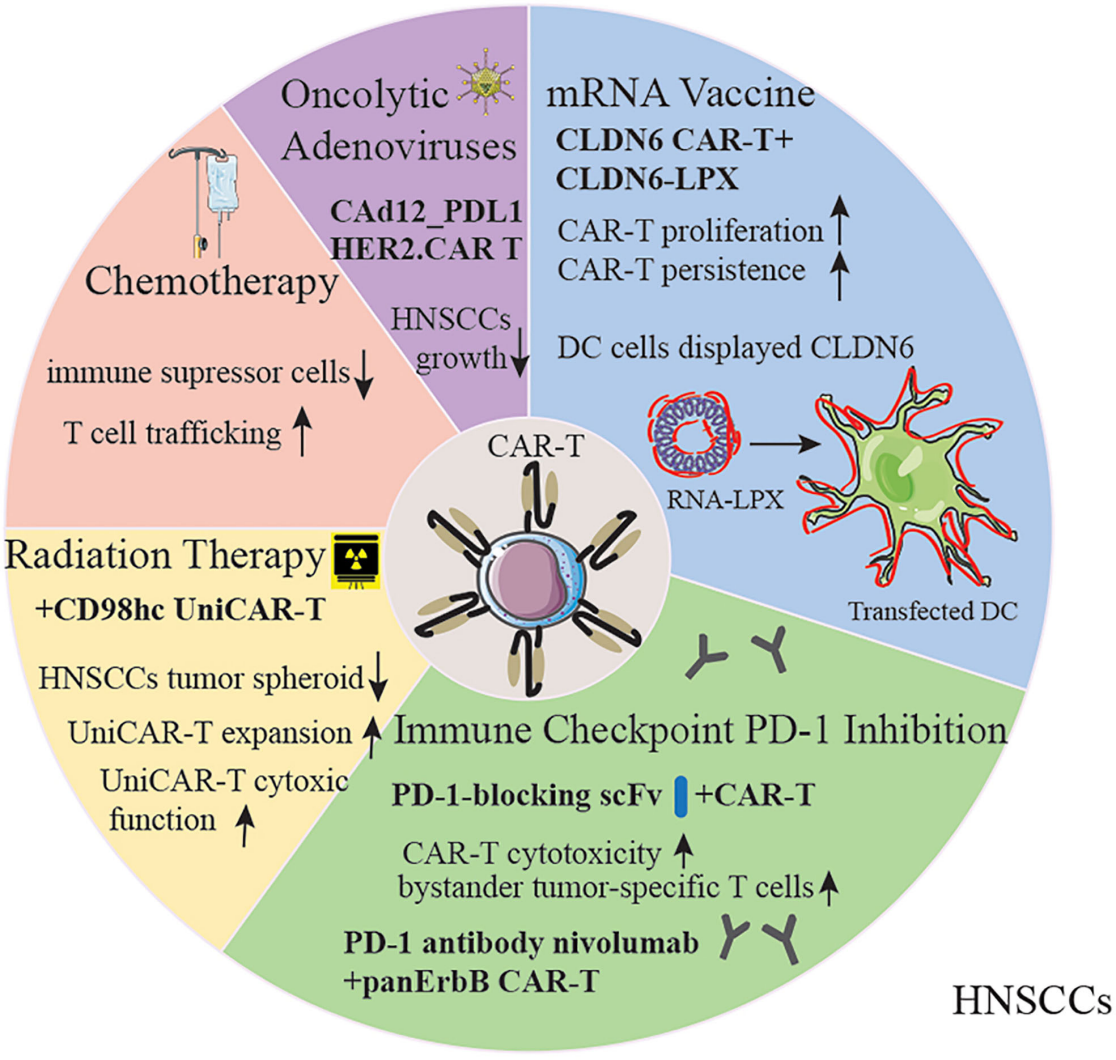
Strategies for improving CAR-T therapy for HNSCC patients. The capability of CAR-T cells could be amplified by chemotherapy, radiation therapy, oncolytic adenoviruses, mRNA vaccine and immune checkpoint inhibitors. DC, Dendritic Cells; RNA-LPX, RNA-lipoplex.

Oncolytic adenoviruses are one of the most employed oncolytic viruses as they can elicit immune-stimulating signals that can substantially eliminate local immunosuppression, cause tumor cell lysis, and improve antitumor immune responses (99). However, limitations such as short persistence, host antiviral immune response, are impeding the utilization of the clinic (100). To overcome them, helper-dependent adenoviral vectors (HDAds) are deleted of all viral genes and can be armed with approximately 34 kb of therapeutic transgenes, which have demonstrated better efficacy for CAR-T immune therapy (101). Further evidence suggests that the feasibility of encoding the PD-L1 blocking antibody and IL-12p70 (CAd12_PDL1) combined with HER2-CAR-T cells infusion in HNSCC xenograft and orthotopic models. Results showed controlled primary and metastasized tumor growth and prolonged survival (102). In addition to this, a phase I trial involving patients with HER2-positive cancer including HNSCCs, utilizing CAdVEC (a genetically modified oncolytic viral strain of human adenovirus (Ad) with potential immunostimulating and antineoplastic activities) coupled with HER2-specific CAR-T cells, is in recruitment (NCT03740256). We have summarized different strategies to enhance the efficacy of CAR-T cell therapy (**Figure 3**).

## 5 Overview of current status of CAR-T clinical trials in HNSCCs

**Figure 4:**
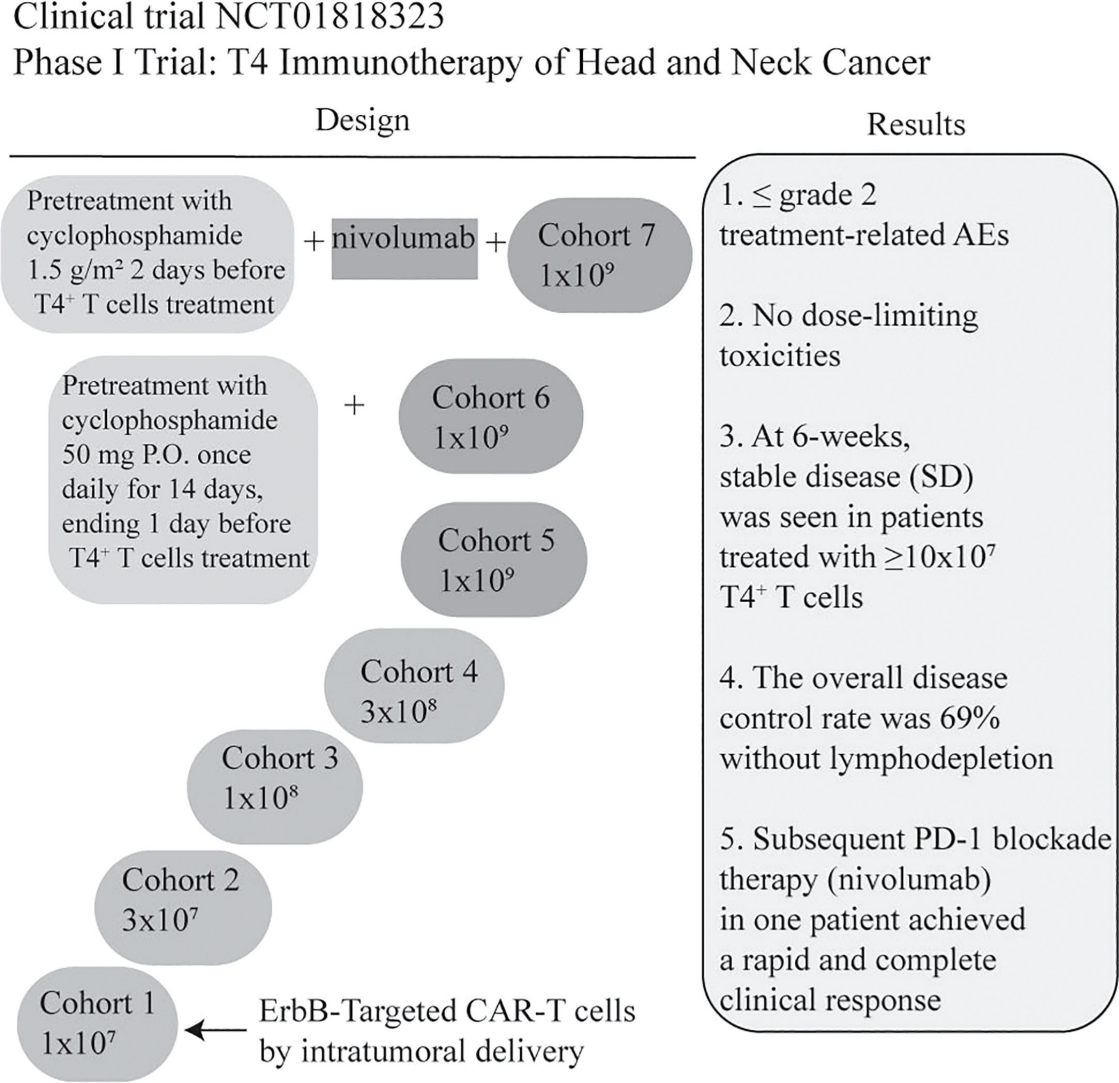
Summary of a Phase I clinical trial of T4 Immunotherapy for head and neck cancer. CAR-T cells co-express T1E28ζ, a promiscuous ErbB ligand coupled to a CD28^+^CD3ζ endodomain, and 4αβ, an IL-4-responsive chimeric cytokine receptor. CAR-T manufacturing was successful in all cases (13/13) even though the rate of lymphopenia was 62%. The dose of CAR T-cell was escalated through 5 cohorts from 1x10^7^ -1x10^9^ administered as a single treatment, by multifocal intra-tumoral injection without lymphodepletion. All patients will be monitored for dose-limiting toxicities (DLT) for 4 weeks before proceeding to the next dose level (24). In addition, the clinical results are illustrated briefly in the figure (25). Treatment-related AEs: Treatment-related adverse events (AEs); nivolumab: anti-PD-1IgG4 antibody; P.O.: per mouth.

NCT01818323 is the first ongoing clinical trial for patients with locally advanced/recurrent HNSCC. T4 immunotherapy is an autologous T cell therapy. A retroviral vector has been used to modify T cells for gene therapy. T cells coexpress two chimeric receptors: T1E28z and 4αβ. T1E28z is a chimeric antigen receptor that engages multiple ErbB dimers commonly overexpressed in HNSCC. On the other hand, previous evidence demonstrated that the addition of IL-4 to T-cells that express 4αβ induced STAT3/STAT5/ERK phosphorylation and exponential proliferation (23). 4αβ, a chimeric cytokine receptor, is therefore designed to insert into T4^+^ T-cells (24). T4 immunotherapy displays antitumor activity against HNSCC cell lines and tumors *in vivo*, without significant toxicity to mice. In this clinical trial, a traditional 3 + 3 dose escalation design is used. If maximum tolerated dose remains undefined, cohorts 6/7 will receive low or high-dose cyclophosphamide before 1x10^9^ T4^+^ T cells infusion. In cohort 7, patients are designed to receive 3 doses of nivolumab before CAR-T treatment (24). However, risk of on-target off-tumor toxicity should be considered due to low-level ErbB expression in normal tissues. To overcome it, a ErbB-targeted CARs capable of detecting the hypoxia of HNSCC might be an option in the future (59). Further investigation on the feasibility of T4 manufacture was evaluated by Papa et al. The results suggested that T4 manufacture was successful, and the overall disease control rate for patients with advanced HNSCC was 69% after T4 immunotherapy without lymphodepletion. In addition, treatment-related adverse effects were ≤ grade 2, without dose-limiting toxicities. One patient achieved a rapid and complete response after PD-1 inhibitor nivolumab treatment (25) (**Figure 4**). Therefore, intra-tumoral administration of T4 in patients with advanced HNSCC could be safe and effective. When HNSCC patients are treated with CAR-T cells by multifocal intra-tumoral injection, lymphodepletion might not be necessary. Conversely, Hirayama et al. reported that the response to lymphodepletion impacts progression-free survival (PFS) in patients with aggressive non-Hodgkin lymphoma treated with CD19 CAR-T cells. Higher intensities of cyclophosphamide and fludarabine lymphodepletion are associated with higher levels of monocyte chemoattractant protein-1 (MCP-1) and IL-7, contributing to better PFS (NCT01865617) (103). However, the evidence of CAR-T for human solid tumors is rare. Suryadevara et al. revealed that CD28-4-1BBz CARs therapy required lymphodepletion to eliminate Tregs cells in murine solid tumor models (104). Inconsistently, Wang et al. generated PD-1 and T cell receptor (TCR) deficient mesothelin-specific CAR-T (MPTK-CAR-T) cells using CRISPR-Cas9 technology and assessed the efficacy of CAR-T in MSLN^+^ patients with advanced solid tumors who received a second or third infusion of MPTK-CAR-T cells without prior lymphodepletion. There was no dose-limiting toxicity or unexpected adverse events. Nevertheless, the approach of using CRISPR-engineered T cells to delete TCR in the study cannot be compared to T4 immunotherapy due to the differences in the CAR-T construct, the type of solid tumor and the delivery method. Whether lymphodepletion impacts CAR-T cell expansion, functionality, long-term persistence and toxicity in the treatment of solid tumors should be further investigated (105).

Most current clinical trials targeting different antigens of HNSCC patients are in recruiting status except for NCI 04249947 and EudraCT 2019-004323-20 are ongoing. Among these clinical trials, patients with HNSCC will receive autologous T cell infusion. However, in NCI 01818323 and NCI 03740256, participants will receive intratumoral injection to reduce the toxicity of CAR-T therapy. Additionally, there is a CAR-γδT (NCT04107142) and CAR-NK cell (NCT04847466) trial targeting HNSCC patients that has been in recruiting status.

Notably, in a phase 1/2a clinical trial conducted in Germany, an RNA vaccine was employed by BioNTech Cell & Gene Therapies GmbH in patients with CLDN6^+^ refractory or relapsed advanced solid tumors (EudraCT No. 2019-004323-20). This study aimed at investigating and assessing the security and initial efficacy of CLDN6 CAR-T cells with or without CLDN6 RNA-lipoplexes (LPX) among the recruited patients. RNA-LPX are composed of liposomes and are widely utilized as *in vitro* transfection reagents. Briefly, this CAR Vaccine is derived from BioNTech´s RNA-LPX technology modified by delivery nanoparticles to APC or negatively charged mRNA, or by shielding positive surface charges with PEG (106, 107). It is an ongoing, multicenter clinical trial involving in our Integrated Oncology Center (CIO) (**Table 1**) and might be a promising strategy for HNSCCs immune therapy in the future.

## 6 Future perspectives

CAR-T cell therapy remains in its infancy although it has been moving beyond its extraordinary success in hematological malignancies. Extensive research in solid tumors revealed that CAR-T cell therapy has tremendous promise in treating refractory and metastatic neoplasms resistant to conventional treatments, including HNSCCs. Recent advances in engineering technologies, novel target antigen spotting, and combination therapies have shown great potential in overcoming hurdles to the development of CAR-T cell therapy in HNSCCs, such as related on-target off-tumor toxicities, the immunosuppressive nature of the tumor microenvironment, and poor T-cell homing to target antigens. Still, further research is necessary before CAR-T cell treatment could be used effectively and safely for people with HNSCCs.

In addition, genetically modifying γδT and NK cells to express CARs has demonstrated encouraging preclinical outcomes and is presently being investigated in a number of clinical studies. More importantly, CIK cells with dual innate and adaptive cytotoxic function have already been modified into CAR-CIK in preclinical solid tumor treatment (108–111) and more effective based on biodistribution and toxicity analyses. Therefore, CAR-CIK might be a new paradigm in solid tumor cellular immunotherapy.

In particular, personalized CAR-T immunotherapy might address the issue of solid tumor heterogeneity. Methodologically, Cafri et al. developed a novel approach using tumor-infiltrating lymphocytes to identify the specific immunogenic mutations expressed in patients’ tumors. They concatenated neoantigens and mutations of driver genes into a single mRNA construct to vaccinate patients with metastatic gastrointestinal cancer. This method provides the possibility of using specific immunogenic mutations in mRNA-LPX or predicted neoepitopes in CAR-T therapy (112). Intriguingly, Miltenyi Biotec B.V. & Co. KG introduced the MICS (MACSima Imaging Cyclic Staining) technology, which identifies hundreds of protein targets derived from a single specimen based on immunofluorescent imaging. Using this technology, we can identify potential target genes derived from individual patients for CAR-T therapy (113). Further advanced methods could be applicable for CAR-T personalized therapy.

## Author contributions

CH, ML YL and YZ wrote the manuscript. CH, ML and YL revised the manuscript. CH, HL and YL reviewed the manuscript and AS provided helpful discussions. IS-W revised and supervised the manuscript. All authors contributed to the article and approved the submitted version.
